# Variation in body condition indices of crimson finches by sex, breeding stage, age, time of day, and year

**DOI:** 10.1093/conphys/cot020

**Published:** 2013-07-16

**Authors:** Olga Milenkaya, Nicole Weinstein, Sarah Legge, Jeffrey R. Walters

**Affiliations:** 1 Department of Biological Sciences, Virginia Polytechnic Institute and State University, Blacksburg, VA 24061, USA; 2 VA-MD Regional College of Veterinary Medicine, Virginia Polytechnic Institute and State University, Blacksburg, VA 24061, USA; 3 Australian Wildlife Conservancy, Mornington Wildlife Sanctuary, Derby, WA 6728, Australia; 4 Research Institute for Environment and Livelihoods, Charles Darwin University, Casuarina, NT 0909, Australia

**Keywords:** Body condition, crimson finch, habitat quality, haematocrit, heterophil to lymphocyte ratio, *Neochmia phaeton*

## Abstract

Body condition indices are increasingly applied in conservation to assess habitat quality, identify stressed populations before they decline, determine effects of disturbances, and understand mechanisms of declines. To employ condition indices in this manner, we need first to understand their baseline variability and sources of variation. Here, we used crimson finches (*Neochmia phaeton*), a tropical passerine, to describe the variation in seven commonly used condition indices by sex, age, breeding stage, time of day, and year. We found that packed cell volume, haemoglobin, total plasma protein, and scaled mass were all significantly affected by an interaction between sex and breeding stage. Furcular fat varied by sex and breeding stage and also trended by year, scaled mass showed a positive trend with age and varied by time of day, and haemoglobin additionally varied by year. Pectoral muscle scores varied and heterophil to lymphocyte ratio trended only by year. Year effects might reflect a response to annual variation in environmental conditions; therefore, those indices showing year effects may be especially worthy of further investigation of their potential for conservation applications. Pectoral muscle scores and heterophil to lymphocyte ratio may be particularly useful due to the lack of influence of other variables on them. For the other indices, the large variation that can be attributed to individual covariates, such as sex and breeding stage, suggests that one should not interpret the physiological condition of an individual as measured by these indices from their absolute value. Instead, the condition of an individual should be interpreted relative to conspecifics by sex, breeding stage, and possibly age.

## Abstract (precis)

Assessing an animal's body condition may be useful in conservation if condition reflects habitat quality. We found that interpretation of most body condition indices can be confounded by effects of breeding stage, sex and age, and only some vary in ways consistent with the idea that they reflect environmental quality.

## Introduction

Body condition is the physiological state of an individual and reflects how successful that individual is in interacting with its environment. Condition is higher when an individual acquires and assimilates more resources (Tomkins *et al.*, 2004), and is usually employed in a relative sense to compare individuals within populations or between places and times. Without a single or direct measure of condition itself, researchers employ body condition indices as tools to measure specific aspects of that physiological state (e.g. nutritional status, stress levels, and immune function), in both basic ecological and applied conservation frameworks.

Conservation physiology is an emerging scientific discipline that applies physiological concepts, tools, and knowledge to conservation in broad ways (Cooke *et al.*, 2013). Body condition and body condition indices are a physiological concept and tool, respectively, with many applications in conservation (Table 1; Stevenson and Woods, 2006). Loss, fragmentation, and degradation of habitat continue on a global scale, and it is critical to biodiversity conservation to assess the effects of changes in habitat on biota. Measuring habitat quality directly is not always feasible, and measuring the condition of individuals offers an alternative method to assess habitat quality (Marra and Holberton, 1998; Johnson, 2007; Homyack, 2010; Ellis *et al.*, 2012). Body condition indices have been used to assess habitat quality, inform habitat management and restoration, identify stressed populations before they decline, determine effects of disturbances, and understand mechanisms of declines (Table 1). They have been used to identify fine-scale habitat requirements that may otherwise be missed by traditional occupancy modelling (Maron *et al.*, 2012) and to provide insights into the nutritional status of animals in different habitat types (Dyer *et al.*, 2009, 2010). Condition indices have been used to assess the effects of habitat fragmentation on a wide range of vertebrates, including birds (Suorsa *et al.*, 2003), mammals (Henry *et al.*, 2007; Johnstone *et al.*, 2012a), and amphibians (Janin *et al.*, 2011).

**Table 1:** Examples of body condition indices being applied in conservation, including those that reflect resource (e.g. food) acquisition and allocation, such as body mass and measures of fat reserves, as well as haematological, biochemical, and endocrine indices that assess other aspects of physiological condition

Conservation application	Condition index	Species	References
Assessing habitat quality	Corticosterone	American redstart (*Setophaga ruticilla*)	Marra and Holberton (1998)
	Fat score, mass, stored fat, haematocrit, H/L ratio, WBC counts, non-estrified fatty acids, glucose, glycerol, uric acid, β-hydroxybutyrate, and triglycerides	Southwestern willow flycatcher (*Empidonax traillii extimus*)	Owen *et al.* (2005)
Assessing habitat requirements	H/L ratio	Eastern yellow robin (*Eopsaltria australis*)	Maron *et al.* (2012)
Assessing nutritional requirements	Mass, WBC counts, packed cell volume, total plasma protein, serum calcium, albumin, serum phosphorus, uric acid, creatine kinase, and aspartate aminotrasferase	Greater sage grouse (*Centrocercus urophasianus*)	Dyer *et al.* (2009, 2010)
Assessing effects of habitat fragmentation	Corticosterone and mass adjusted for structural body size	Eurasian treecreeper (*Certhia familiaris*)	Suorsa *et al.* (2003)
	Haematocrit	Bats: *Artibeus jamaicensis* and *Artibeus obscurus*	Henry *et al.* (2007)
	Haemoglobin, haematocrit, N/L ratio, WBC counts, mean red blood cell volume, mean cell haemoglobin content, mean cell haemoglobin concentration, and red blood cell distribution width	Agile antechinus (*Antechinus agilis*)	Johnstone *et al.* (2012a)
	Corticosterone and mass adjusted for structural body size	Common toad (*Bufo bufo*)	Janin *et al.* (2011)
Assessing effects of ecotourism	Haematocrit, leucocrit, total serum protein, WBC differentials, and total antioxidant capacity to total oxidative status ratio	Southern stingray (*Dasyatis americana*)	Semeniuk *et al.* (2009)
	Corticosterone	Magellanic penguin (*Spheniscus magellanicus*)	Walker *et al.* (2005)
	Corticosterone and mass adjusted for structural body size	Yellow-eyed penguin (*Megadyptes antipodes*)	Ellenberg *et al.* (2007)
Assessing stress due to logging activity	Corticosterone	Northern spotted owl (*Strix occidentalis caurina*)	Wasser *et al.* (1997)
Assessing effects of oiling on wildlife	Haemoglobin	River otter (*Lontra canadensis*)	Ben-David *et al.* (2002)

For a review of body condition indices used in conservation broadly, see Stevenson and Woods (2006), and for reviews of the use of body condition indices to assess habitat quality in particular, see Johnson (2007), Ellis *et al.* (2012), and Homyack (2010). Abbreviations: H/L ratio, heterophil to lymphocyte ratio; N/L ratio, neutrophil to lymphocyte ratio; and WBC, white blood cell.

Condition indices have also been used to assess impacts of stressors and disturbances other than habitat alteration (Table 1). Condition indices were employed, for example, to detect deleterious effects of ecotourism on wildlife (Walker *et al.*, 2005; Ellenberg *et al.*, 2007; Semeniuk *et al.*, 2009), of the presence of logging roads and nearby timber harvesting on northern spotted owls (*Strix occidentalis caurina*; Wasser *et al.*, 1997), and of oiling on sea otters even after they were treated (*Lontra canadensis*; Ben-David *et al.*, 2002). Other applications of condition indices in conservation are in testing hypotheses about the agents of population decline (e.g. Owen *et al.*, 2005) and elucidating the mechanisms of declines (Maute, 2011). Finally, Cooke *et al.* (2013) encourage us to move beyond the identification of problems and towards the constructive use of this knowledge in providing conservation solutions.

Use of condition indices in conservation is hampered by the paucity of data on their baseline variability and sources of variation. Despite their frequent use, condition indices are poorly described for many species in the wild and, in particular, for those from the tropics (Stevenson and Woods, 2006). The covariates that may affect these indices are even less well documented, and understanding of this variability is needed to inform our interpretation of condition indices (Dawson and Bortolotti, 1997; Fair *et al.*, 2007).

Likely sources of variation that may affect condition indices include sex, age, breeding stage, time of day, and annual and seasonal variation in the environment. While the last two parameters represent the sort of interaction between environment and individual that reflects uses of these indices in conservation, the first four parameters represent variables that potentially confound these uses. Condition indices may vary between males and females because of influences of sex hormones (Shallin Busch *et al.*, 2011) and because of differences in sex-specific workload, especially during breeding (Hõrak *et al.*, 1998b). Age may affect condition indices if individuals experience physiological senescence, have variable exposure to immune challenges with age, or allocate their resources differently with age. Condition indices may vary across the different stages of the breeding cycle because of hormonal changes and different energy requirements associated with these stages (Williams *et al.*, 2004; Fair *et al.*, 2007). Finally, time of day is known to affect the mass and fat stores of diurnal birds due to overnight fasting (Pravosudov and Grubb, 1997; Sepp *et al.*, 2010).

This study is part of a larger research project, the objectives of which are to assess the consequences of different land-management regimens on wildlife physiology and to test whether condition indices predict fitness such that they can inform habitat management. The habitats under consideration are northern Australia's tropical savanna and interstitial areas embedded within this matrix, including riparian habitats, all of which are threatened by non-native herbivores (cattle, horses, donkeys, buffalo, and pigs) and inappropriate fire regimens (Franklin *et al.*, 2005; Woinarski *et al.*, 2011). Broadly, we are examining the impacts of differing fire and introduced herbivore management on the persistence and physiology of native species, and more specifically, we are investigating whether condition indices can be used to provide prior warning of impending species declines. To examine this, we are comparing condition indices in relationship to the presence/absence of introduced herbivores, and to whether fire is managed (small-scale and low-intensity fires) or unmanaged (frequent, extensive, and high-intensity fires).

Here, we take the first step towards assessing the impact of land-management regimens on wildlife physiology by reporting baseline condition indices sampled from a native passerine in habitat that has been managed for conservation. We then explore the variability of these indices by sex, breeding stage, age, time of day, and year. Our goal in describing this variation is to inform our interpretation of condition indices as conservation tools when comparing across populations.

## Materials and methods

### Species description and general methods

Crimson finches (*Neochmia phaeton*, Family Estrildidae) are restricted to riparian zones throughout the tropical savannas of northern Australia and New Guinea (Higgins *et al.*, 2006). Two subspecies of crimson finches are recognized, *N. p. phaeton* and *N. p. evangelinae* (Higgins *et al.*, 2006); the latter includes populations in southern Papua New Guinea and four populations on the Cape York peninsula, Australia and is listed as vulnerable in the Commonwealth of Australia (Dorricott and Garnett, 2007) and as near threatened internationally (Garnett *et al.*, 2011). The main threats to *N. p. evangelinae* in Australia include fire regimens and invasive plant species that alter the vegetation structure used by the finches (Dorricott and Garnett, 2007). In contrast, *N. p. phaeton* is widely distributed throughout northern Australia and is listed as being of least concern (Garnett *et al.*, 2011). *Neochmia phaeton phaeton* is not as threatened as its congener, but it is a riparian habitat specialist and is therefore susceptible to habitat fragmentation caused by land-management practices such as burning and grazing.

*Neochmia phaeton phaeton* are small (females 9.7 g and males 10.0 g), sedentary passerines that breed during the wet season (roughly December–April in our study area; Milenkaya *et al.*, 2011). They breed as socially monogamous pairs, in which the male builds the nest, the female lays a clutch of an average of five eggs, and both sexes incubate the eggs, and provision and defend the young (Milenkaya *et al.*, 2011). Detailed breeding biology and life-history traits of crimson finches from our study area are described by Milenkaya *et al.* (2011).

We monitored a population of *N. p. phaeton* at the Australian Wildlife Conservancy's Mornington Wildlife Sanctuary in northwest Australia (17°30′49″ S, 126°06′39″ E). The study area was a 2 km stretch of riparian habitat along Annie Creek that is actively managed for conservation. Here we monitored the crimson finch population for breeding activity and sampled the individuals for condition indices over four consecutive breeding seasons (from 15 November 2006 to 11 April 2007, from 5 December 2007 to 31 May 2008, from 9 December 2008 to 29 May 2009, and from 10 December 2009 to 10 May 2010).

We captured adult crimson finches in mist-nets during the morning (up to 6 h) to minimize time-of-day effects on condition indices (Norte *et al.*, 2009b; Sepp *et al.*, 2010). We banded the birds with a metal band (Australian Bird and Bat Banding Scheme) and up to three plastic colour bands for individual recognition. We determined the sex of each bird based on their sexually dimorphic plumage (Higgins *et al.*, 2006). To sample for haematological condition indices, we punctured the birds' brachial vein with a needle and collected whole blood (up to 40 µl) into plastic, sodium-heparinized micro-haematocrit capillary tubes. We plugged the tubes and stored them on ice until further processing (within several hours; see subsection Condition indices below).

We sampled 372 individual birds, which we aged based on known hatch dates, juvenal plumage, and molt limits. We were able to assign an accurate age to the individuals that were originally banded as nestlings or juveniles (25%, *n* = 92). Birds that were in adult plumage upon first capture were aged as being in their first year (23%, *n* = 85) if they had a molt limit between their retained, juvenal primary coverts and adjacent newer feathers (Milenkaya *et al.*, 2011), and in at least their second year if they had newly molted and fresh primary coverts at the beginning of the breeding season (10%, *n* = 39). Together, we refer to these individuals as known-aged birds. In contrast, all other individuals could not be aged because they were first captured as adults, and data on their molt were not collected (42%, *n* = 156).

We monitored the breeding attempts of individual birds closely and could therefore attribute captured birds as being in one of the following stages of the breeding cycle at the time of capture: pre-breeding, nest building, egg laying, incubating, nestling, or post-breeding. Crimson finches in our study area are multi-brooded (Milenkaya *et al.*, 2011), so the nest building, egg laying, incubating, and nestling stages occur throughout the breeding season and do not necessarily represent the first breeding attempt of the season. The pre- and post-breeding stages, however, correspond to the beginning and end of the breeding seasons, respectively. Individuals were considered to be in the post-breeding stage when it was 3 weeks or more after their final breeding attempt ended (either through nest failure or because the last set of young became independent at 3 weeks post-fledging).

### Condition indices

Mass corrected for size reflects nutrient stores (Brown, 1996) and is perhaps the most common measure of condition. Along with fat and muscle scores, these data are minimally invasive to collect and are therefore often used in field studies. Fat scores correspond to energy reserves, while muscle scores indicate protein as well as energy reserves. We weighed birds using a 30 g spring scale to 0.1 g and measured the distance from the tip of the beak to the back of the head using callipers as a measure of structural size. We did not measure tarsus length because measuring it precisely and consistently is challenging (Pyle, 1997), especially on a small passerine, such as the crimson finch. We used the mass and head–beak measurement to calculate a scaled mass index (scaled mass) as a measure of mass corrected for size, following Peig and Green (2009). We calculated scaled mass separately for males and females because they are sexually dimorphic in size (Milenkaya *et al.*, 2011). Finally, we scored the amount of pectoral muscle around the keel bone on a 0–3 scale (muscle score) and the amount of fat in the furcular hollow on a 0–4 scale (fat score; Table 2).

**Table 2:** Scoring criteria for furcular fat and pectoral muscle

Score	Furcular fat	Muscle
0	No fat observed	No pectoralis observed
1	Fills <33% of furculum	Keel is very prominent, with minimal pectoralis
2	Fills 34–66% of furculum	Pectoralis is clear, but does not exceed the keel
3	Fills 67–99% of furculum	Pectoralis exceeds keel
4	Fat is flush with furculum	–

Other readily available condition indices include haematological parameters that are a result of an individual's nutritional status, stress level, and immune function. Packed red blood cell volume (PCV) is the proportion of red blood cells to the total volume of blood, and is often considered an aggregate indicator of overall health (e.g. Garvin *et al.*, 2007). Low PCV and anaemia (PCV <35% in birds; Campbell and Ellis, 2007) may be caused by blood loss (e.g. parasitism; Szabó *et al.*, 2002), by destruction of erythrocytes (e.g. infectious agents or toxins), or by decreased production of erythrocytes [e.g. disease, nutritional stress (Harrison and Harrison, 1986, but see Fair *et al.*, 2007), or toxin exposure (Campbell and Ellis, 2007)]. High PCV may be due either to dehydration or to an increased energy demand and resultant need for increased oxygen-carrying capacity (Carpenter, 1975; Hõrak *et al.*, 1998a). The relationship between PCV and the oxygen-carrying capacity of blood is parabolic, such that both very low and very high PCV values result in decreased oxygen transport capability (Birchard, 1997). Packed cell volume is related to haemoglobin concentration (Velguth *et al.*, 2010), which also reflects the oxygen-carrying capacity of the blood (Birchard, 1997) and is lower in clinically sick birds compared with healthy birds (Averbeck, 1992). Before collecting blood with a capillary tube, we collected ∼5 µl of it in a Hemacue cuvette and used the portable HemoCue Hb 201+ Analyzer (HemoCue, Inc., Cypress, CA, USA) in the field to estimate haemoglobin concentration (haemoglobin) from whole blood. Within a few hours of collecting blood in capillary tubes, we centrifuged (Hettich Haematokrit 210) the tubes for 15 min at 1433.6*g* and read the proportion of red blood cells to the total volume of blood (packed cell volume).

Total plasma protein is a measure of proteins present in plasma; primarily albumin and globulin. Dehydration causes increases in both albumin and globulin, but an increase in globulin alone suggests inflammation and immune system stimulation (Rosenthal, 2000), and some have found that overall total plasma protein increases with inflammation/infection (de Lope *et al.*, 1998; Ots and Hõrak, 1998). Decreases in total plasma protein may be caused by parasites (Norte *et al.*, 2009a), and poor nutrition may result in decreased albumin (Rosenthal, 2000), suggesting that albumin levels may reflect diet. Total plasma protein is therefore sometimes interpreted as an indicator of nutritional status (Gavett and Wakeley, 1986; Brown, 1996) and as a measure of body condition (e.g. Schoech and Bowman, 2003). During the 2008–09 and 2009–10 field seasons, we extracted the plasma from the capillary tube after centrifugation and read the total plasma protein value (in grams per decilitre) with a hand-held refractometer (HR-200 ATC refractometer; AFAB Enterprises, Eustis, FL, USA). The total plasma protein values for bird samples obtained with this technique are sometimes consistent with and sometimes higher than values of those obtained from chemical analyses (George, 2001).

Heterophils and lymphocytes are white blood cells primarily involved in the immune response (Campbell and Ellis, 2007). A high heterophil to lymphocyte ratio (H/L ratio) suggests an active inflammation response (Campbell and Ellis, 2007) and can be correlated with elevated corticosterone levels in response to chronic stress (Davis *et al.*, 2008). We sampled for H/L ratio only during the 2008–09 and 2009–10 field seasons. During these years, we prepared blood smears immediately from collected blood and fixed the slides in 100% methanol on the same day. We then stained the smears with Dip Quick Stain Set (Jorgensen Laboratories, Inc., Loveland, CO, USA) at a later date. One of us (O.M.) performed a leucocyte differential count under ×1000 magnification by identifying 100 leucocytes as a heterophil, lymphocyte, eosinophil, basophil, or monocyte. The H/L ratio was calculated as the relative proportion of heterophils to lymphocytes counted in the blood smear.

### Statistical analyses

We used all sampled individuals to describe the distribution (mean ± SD, range) of each condition index and to test for pairwise correlations between the indices. If an individual was sampled more than once, we used the average value for that individual for each condition index in these descriptive analyses. We tested whether the continuous variables [packed cell volume, haemoglobin, total plasma protein, scaled mass, and log(H/L ratio)] were correlated with one another by using Pearson's correlation (cor function in R version 2.14.1; R Development Core Team, 2012). We tested whether these continuous indices are correlated to muscle and fat scores, and whether muscle and fat scores are correlated to each other, by using polyserial and polychoral correlations, respectively (package polycor in R version 2.14.1; Fox, 2010).

To test the effects of sex, age, year, time of day, and breeding stage, as well as the interaction between sex and breeding stage, on each condition index we used restricted maximum likelihood to fit linear regression models. For these analyses, we included only known-aged individuals in the data set. To determine normality, we used the Shapiro–Wilk test and visual examination of the model residuals. The H/L ratios were base e log transformed to fit the assumptions of normality, whereas other condition indices were normally distributed. We included individual as a random effect in each of these models because some individuals were sampled more than once. To determine whether this analysis missed any significant effects due to small sample size, we conducted a second analysis in which all individuals were included, regardless of whether they were of known age or not. We calculated the time of day of capture as the length of time (in minutes) since sunrise.

After finding significant effects of year and of the interaction between sex and breeding stage on many condition indices (see Results), we examined the parameter estimates and inferred statistical significance from the 95% confidence intervals for those estimates to better understand the effects of these terms on the condition indices. We set statistical significance at α = 0.05. Our figures show least-square means (±SEM) which are consistent with the raw data. We ran all models in JMP 9.0 (SAS Institute Inc., Cary, NC, USA).

## Results

We sampled 372 individual birds for some or all of the condition indices during the 4 year study, of which 40% were sampled once (*n* = 149), while the rest were sampled more than once, as follows: twice, 25% (*n* = 94); three times, 13% (*n* = 49); four times, 10% (*n* = 37); five times, 5% (*n* = 20); six times, 4% (*n* = 15); and seven times or more, 2% (*n* = 7). The distributions of observed values (means ± SD, minimum, maximum) for each condition index are summarized in Table 3. Of the pairwise correlations among condition indices, only one correlation was strong and significant, that of packed cell volume to haemoglobin (*r* = 0.74, *P* < 0.0001; Table 4).

**Table 3:** The distribution of observed values for each condition index among crimson finches, including the mean, standard deviation, minimum, maximum, and sample size (*n*)

Condition index	Mean	SD	Minimum	Maximum	*n*
Packed cell volume (%)	48.5	2.92	40	58	322
Haemoglobin (g/l)	174.4	12.81	142	224	335
Total plasma protein (g/dl)	4.93	0.62	3.25	7.2	186
Heterophil to lymphocyte ratio	0.835	0.96	0.10	8.9	149
Scaled mass (g)	9.96	0.72	7.67	12.16	362
Muscle score	2.09	0.54	0.5	3	362
Fat score	2.06	0.93	0	4	362

**Table 4:** Pairwise correlations between condition indices, including the correlation coefficient, the sample size in parentheses, and asterisks indicating significance after Bonferroni correction for the Pearson's correlations (**P < 0.001 and ***P < 0.0001)

	Packed cell volume	Haemoglobin	Total plasma protein	Scaled mass	Log(H/L ratio)	Muscle score	Fat score
Packed cell volume	1	0.74 (310)***	−0.18 (185)	−0.07 (320)	−0.02 (144)	−0.01 (317)	−0.08 (317)
Haemoglobin	–	1	−0.10 (178)	−0.09 (331)	−0.02 (144)	−0.12 (335)	−0.14 (335)
Total plasma protein	–	–	1	0.25 (186)**	−0.02 (144)	0.08 (185)	0.34 (185)
Scaled mass	–	–	–	1	0.00 (149)	0.11 (356)	0.34 (356)
Log(H/L ratio)	–	–	–	–	1	−0.10 (149)	−0.01 (149)
Muscle score	–	–	–	–	–	1	0.18 (362)
Fat score	–	–	–	–	–	–	1

Correlations between a continuous variable [packed cell volume, haemoglobin, total plasma protein, scaled mass, and log(H/L ratio)] and either muscle score or fat score are polyserial correlations, the correlation between muscle score and fat score is a polychoral correlation, and neither are tested for significance.

**Figure 1:**
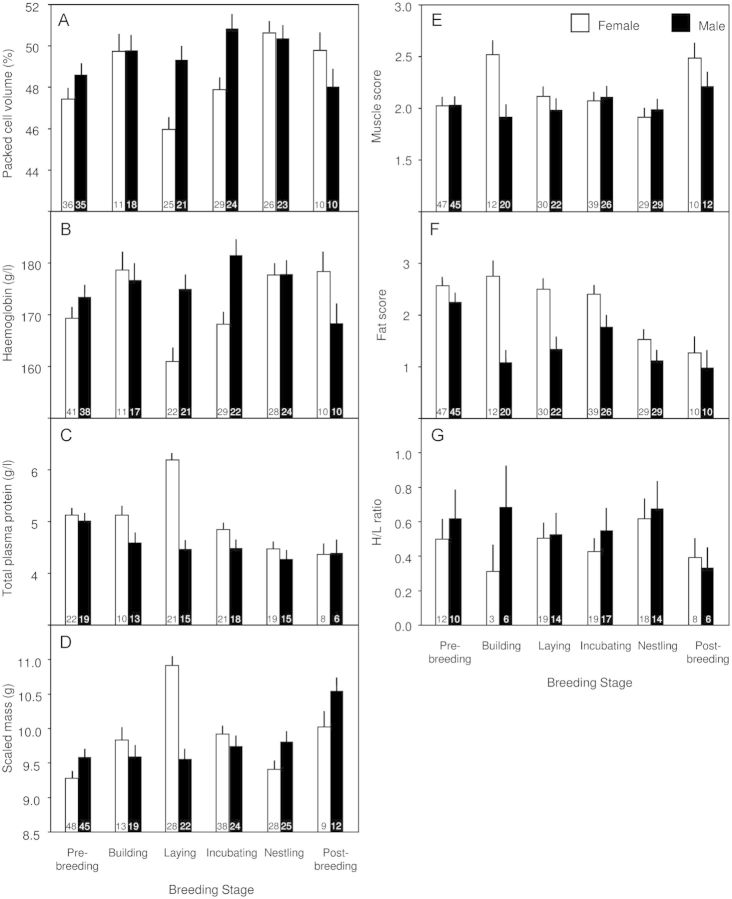
The variation in condition indices among crimson finches by sex and breeding stage. Least-squares means (±SEM) of each condition index by sex and breeding stage, including packed cell volume (**A**), haemoglobin (**B**), total plasma protein (**C**), scaled mass (**D**), muscle score (**E**), fat score (**F**), and heterophil to lymphocyte ratio (H/L ratio; **G**). Sample sizes are given within each bar.

Here we present the results of the regression analysis in which only known-aged individuals were included because these results are consistent with the analysis in which all individuals, whether they were of known age or not, were included. With the exception of H/L ratio, all condition indices varied by an interaction between sex and breeding stage (although muscle and fat score trends were not significant after Bonferroni correction; Table 5). Packed cell volume did not vary among males by breeding stage, but was lower among females than among males during the laying and incubating stages, and was comparable during the other stages of the breeding cycle (Fig. 1A). Packed cell volume was lowest for egg-laying females and highest in the incubating (males only) and the nestling stages (both sexes; Fig. 1A). Haemoglobin followed similar patterns to PCV between the sexes and across the breeding stages (Fig. 1B). Total plasma protein was elevated among egg-laying females and was lower in the nestling stage than in the pre-breeding stage for each sex (Fig. 1C). Females were heavier for their size than males during the egg-laying stage due to egg mass, and were comparable to males during the other breeding stages. Both sexes trended towards a gain in scaled mass during the post-breeding stage (Fig. 1D). Male muscle did not vary significantly between the breeding stages, but females had high muscle during the nest-building stage, the lowest during the nestling stage, and then high again during the post-breeding stage (Fig. 1E). Males and females had similar fat reserves during the pre-breeding stage, and while females maintained high fat until the nestling stage, males decreased in fat during the nest-building stage (Fig. 1F). Fat scores remained low in the post-breeding stage for both sexes (Fig. 1F). Heterophil to lymphocyte ratios did not significantly vary by sex, breeding stage, or the interaction between sex and breeding stage (Fig. 1G and Table 5).

**Table 5:** The variation of each condition index by sex, year, age, breeding stage, sex by breeding stage interaction, and time of day, including the sample size (*n*) and r^2^ for each model, and the degrees of freedom (d.f.), F-ratio (*F*) and *P*-value, with an asterisk denoting significance after Bonferroni correction for each covariate in the model

Condition index covariates	*n*	*r*^2^	d.f.	*F*	*P*-value
Packed cell volume (%)	268	0.70			
Sex			1	4.06	0.05
Year			3	1.88	0.13
Age			4	1.37	0.25
Stage			5	7.63	<0.0001*
Sex × stage			5	4.98	0.0002*
Time of day			1	0.96	0.33
Haemoglobin (g/l)	273	0.70			
Sex			1	2.84	0.09
Year			3	7.13	0.0001*
Age			4	0.94	0.44
Stage			5	4.91	0.0003*
Sex × stage			5	5.39	0.0001*
Time of day			1	1.23	0.27
Total plasma protein (g/dl)	187	0.71			
Sex			1	18.35	<0.0001*
Year			1	0.93	0.34
Age			4	1.70	0.15
Stage			5	12.40	<0.0001*
Sex × stage			5	9.75	<0.0001*
Time of day			1	0.22	0.64
Log(H/L ratio)	146	0.61			
Sex			1	1.71	0.19
Year			1	9.86	0.002
Age			4	0.95	0.44
Stage			5	1.50	0.20
Sex × stage			5	0.58	0.72
Time of day			1	1.03	0.31
Scaled mass (g)	310	0.67			
Sex			1	0.76	0.38
Year			3	1.56	0.20
Age			4	2.49	0.04
Stage			5	13.14	<0.0001*
Sex × stage			5	14.38	<0.0001*
Time of day			1	12.16	0.0006*
Muscle score	320	0.55			
Sex			1	4.58	0.03
Year			3	26.93	<0.0001*
Age			4	0.58	0.68
Stage			5	3.06	0.01
Sex × stage			5	2.75	0.02
Time of day			1	0.88	0.35
Fat score	318	0.29			
Sex			1	29.10	<0.0001*
Year			3	4.96	0.002
Age			4	0.58	0.68
Stage			5	9.35	<0.0001*
Sex × stage			5	2.78	0.02
Time of day			1	2.87	0.09

**Figure 2:**
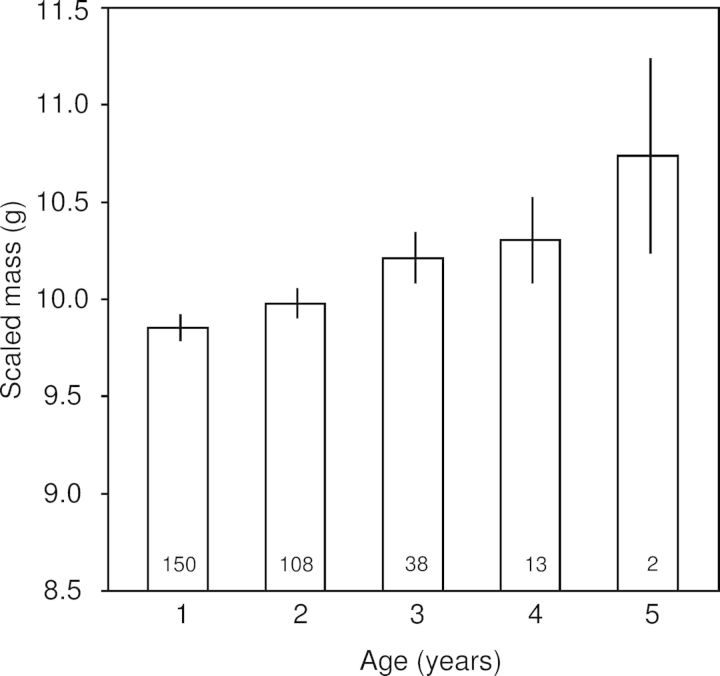
The variation in scaled mass among crimson finches by age. Least-squares means (±SEM) of scaled mass (in grams) by age. Scaled mass trended positively with age, but the variable age was no longer significant in the model after Bonferroni correction. Sample sizes are given within each bar. Other condition indices did not vary significantly by age and are not illustrated here.

Scaled mass was positively correlated with age, and this trend appears to be linear (Fig. 2); however, scaled mass no longer varied significantly by age after Bonferroni correction (Table 5). No other condition indices varied by age (Table 5).

**Figure 3:**
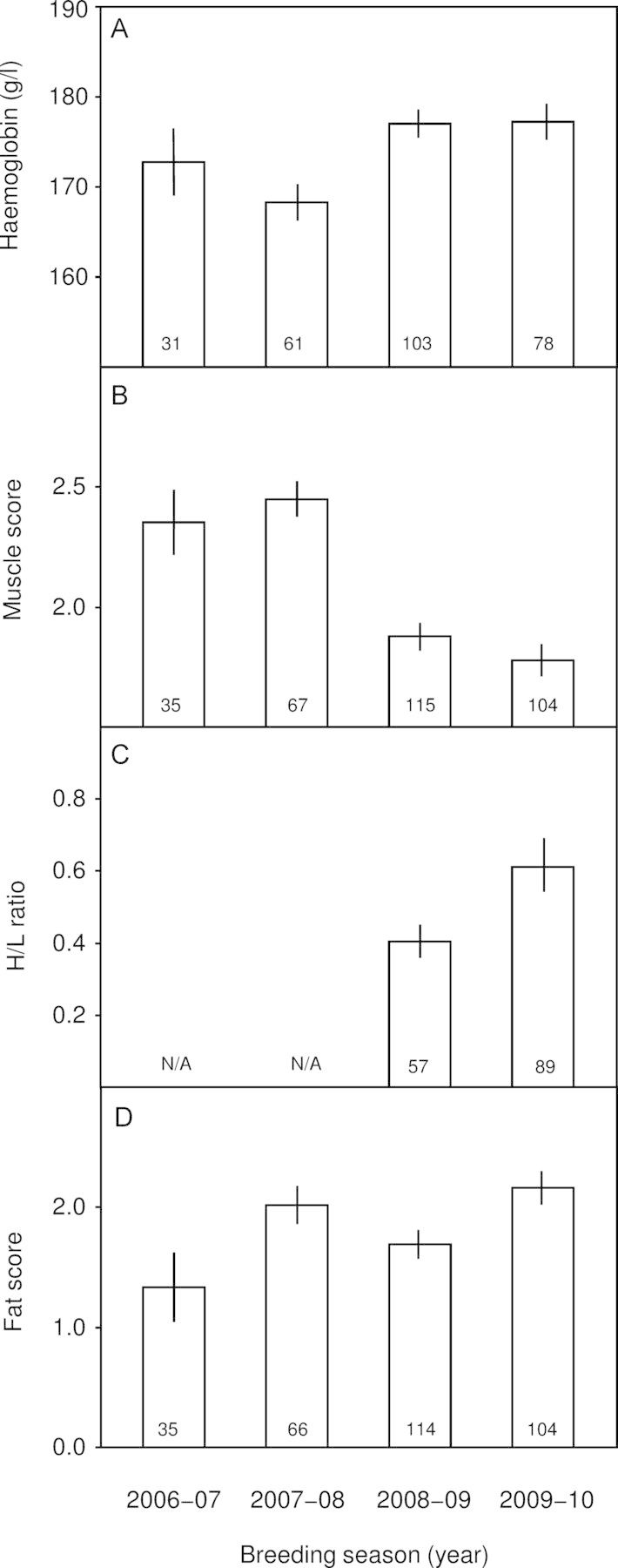
Annual variation in haemoglobin, muscle, H/L ratio, and fat score among crimson finches. Least-squares means (±SEM) of haemoglobin (**A**), muscle score (**B**), H/L ratio (**C**), and fat score (**D**) by year. We include the H/L ratio and fat score here because they trended with year (both *P*-values = 0.002) even though these trends were not significant after Bonferroni correction. The H/L ratio values (C) were back transformed from a base e log to a linear scale. Sample sizes are given within each bar. Other condition indices did not significantly vary by year and are not illustrated here.

We found annual variation among haemoglobin, muscle, H/L ratio, and fat, although the trend for the latter two indices (both *P*-values = 0.002) was no longer significant after Bonferroni correction (Table 5 and Fig. 3). Haemoglobin was lowest during the 2007–08 breeding season (Fig. 3A), muscle was significantly lower in 2008–09 and 2009–10 than during the other two breeding seasons (Fig. 3B), H/L ratios trended lower in 2008–09 compared with the 2009–10 breeding season (Fig. 3C), and fat scores trended lower in the 2006–07 and 2008–09 breeding seasons compared with the other two seasons (Fig. 3D). Scaled mass was the only condition index to vary with time of day (Table 5).

## Discussion

We describe condition indices for a small tropical granivore, the crimson finch, and demonstrate that these indices vary by sex, breeding stage, and to a lesser extent age, year, and time of day. Our results demonstrate that researchers should control for these sources of variation (statistically or via sampling design) to interpret differences in condition correctly between individuals and populations.

### Effects of egg laying by females

We found that most of the condition indices varied significantly by an interaction between sex and breeding stage. For females, much of this effect is most parsimoniously explained as a consequence of egg laying. Packed cell volume and haemoglobin decrease while total plasma protein and scaled mass increase in egg-laying females. This pattern has been reported previously (packed cell volume, Fair *et al.*, 2007; and total plasma protein, Hõrak *et al.*, 1998a; Saino *et al.*, 2001; Wagner *et al.*, 2008). The nadir for packed cell volume among egg-laying females is probably caused by changes in hormone levels (Wagner *et al.*, 2008), and this decrease in the proportion of red blood cells, and subsequently of haemoglobin, is not sufficiently low to be considered anaemia (Campbell and Ellis, 2007). The high total plasma protein levels among egg-laying females may reflect female investment in their offspring's immunity by their transfer of immunoglobulins to the eggs (Saino *et al.*, 2001), but this hypothesis should be tested. Finally, the increased scaled mass among egg-laying females is an artifact of the developing egg mass and clearly does not reflect improved body condition. Generally, the changes in condition indices during egg laying for a given female cannot be interpreted as changes in her condition reflective of her interaction with the environment; however, a comparison of the magnitude of change between females in different environments may be informative in some cases.

### Packed cell volume and haemoglobin

Consistent with the result that packed cell volume and haemoglobin are positively and strongly correlated in crimson finches (Table 4) and in birds generally (Velguth *et al.*, 2010), we found that the interaction between sex and breeding stage was very similar between these two indices. Males did not vary in either index with breeding stage, while females did. Their egg-laying decrease persisted through the incubating stage, when females still had lower values than males; however, female values increased thereafter and were again comparable to males during the nestling and post-breeding stages. A similar pattern was found among mountain white-crowned sparrows (*Zonotrichia leucophrys oriantha*; Morton, 1994).

The interaction between sex and breeding stage may explain the inconsistent results of studies that considered only sex, but did not consider both sex and breeding stage. Some studies found males to have higher PCV values than females (Saino *et al.*, 1997; Owen *et al.*, 2005), while another found females to have higher values (Kilgas *et al.*, 2006a), and still others found no sex difference (Dawson and Bortolotti, 1997; Kasprzak *et al.*, 2006; Hatch *et al.*, 2010). In a meta-analysis of previously published articles, Fair *et al.* (2007) concluded that PCV was not different between the sexes. We suggest that the sexes may vary in their packed cell volume and haemoglobin values, but that this difference is stage dependent. We would not have found a sex effect had we not tested for the interaction term between sex and breeding stage, because the main effect of sex was not significant in our models.

### Total plasma protein

Significant decreases in protein concentration from the pre-breeding to the nestling stage occurred in both sexes, consistent with the findings of Hõrak *et al.* (1998a) (but see Saino *et al.*, 2001). The low total plasma protein levels in females during the nestling stage may be due to depleted protein levels following egg production, but this does not explain why males also have their lowest total plasma protein levels during this stage, suggesting that a different mechanism is involved. Breeding birds may be suppressing their immune function (Greenman *et al.*, 2005) and thereby causing a decrease in total plasma protein levels during the nestling stage (Ardia, 2005). However, this condition index is also influenced by other factors. Total plasma protein levels increase with the nutritional value of food (Gavett and Wakeley, 1986; Brown, 1996), so the variability in total plasma protein among crimson finches may reflect the variability of food resources across the breeding season (e.g. Hõrak *et al.*, 1998a). Total plasma protein levels also increase with disease through an increase in circulating globulins (Ots and Hõrak, 1998), as well as with dehydration. Dehydration is unlikely to be a factor among crimson finches, because they inhabit riparian areas where they have ready access to water. The changes between the pre-breeding and nestling stages we documented may therefore be interpreted as reflecting either changes in diet or changes in immune challenges, response, and/or function. The interpretation of total plasma protein levels in ecological studies is complicated by the fact that several factors can impact this index, and disentangling the mechanism behind a given trend is challenging. For example, high total plasma protein levels may be attributed to an individual being in good condition due to good nutrition, to an individual being in poor condition due to disease, or to dehydration. The lack of a clear and consistent interpretation of total plasma protein suggests that it should be interpreted with caution.

### Scaled mass, fat, and muscle

Body mass is known to increase with time of day in diurnal birds due to overnight fasting (Pravosudov and Grubb, 1997; Sepp *et al.*, 2010), and we found the same among crimson finches, although fat and muscle scores did not change with time of day.

Both sexes had lower fat scores while breeding compared with the pre-breeding stage, but the timing of fat loss differed between the sexes, with males losing their fat during the nest-building stage, whereas females maintained high fat levels until the nestling stage. Crimson finch males, and not females, build the nests (Milenkaya *et al.*, 2011). This activity may explain the loss of fat among males during this stage, either because it is energetically costly or because it is adaptive to maintain lower fat stores during a period of high mobility. Crimson finch females not only had less fat but also had their lowest muscle scores during the nestling stage, suggesting that this is the most challenging time of the breeding season for them. Similar results were found among chick-rearing black-legged kittiwakes (*Rissa tridactyla*; Kitaysky *et al.*, 1999), American kestrels (*Falco sparverius*; Dawson and Bortolotti, 1997), and Savi's warblers (*Locustella luscinioides*; Neto and Gosler, 2009). However, male and female crimson finches provision the young to an approximately equal extent (personal observation by Olga Milenkaya, Virginia Tech), and males did not lose any muscle, fat, or scaled mass during the nestling stage, suggesting that more than simply the cost of provisioning young is involved in the changes we observed in females at this stage.

The optimal body mass of a bird is influenced by physiological and ecological trade-offs between essential activities. The trade-off between starvation and predation (McNamara and Houston, 1990) is an example; fat reserves decrease starvation risk but may increase predation risk, while pectoral muscle reserves lower predation risk through increased flight performance (reviewed by Rogers, 2005). Food availability and predictability, in conjunction with predatory pressure and physiological demands such as thermoregulation, migration, and molt, may all impact optimal mass and the optimal allocation of resources between fat and muscle reserves. All of these variables may change throughout the breeding season of crimson finches, which may in turn affect optimal levels of scaled mass, fat, and muscle. Unfortunately, most of what we know about adaptive mass and its associated trade-offs, and the role of fat and muscle, comes from the study of migrants and northern-hemisphere temperate species, with little being known about sedentary birds in the tropics, such as crimson finches. We suggest that if fat and muscle scores are to be used as indicators of condition among sedentary and tropical birds, then we need to understand the costs and benefits of these reserves better for species that do not face the challenge of migration and that live in climates that are less variable or thermally challenging.

### Sex, age, and year effects

Most of the condition indices we measured also varied significantly by sex. The mechanism behind the variation due to sex is important for interpreting condition because if, for example, this variation is solely due to hormones during the breeding season then sex may be a less important covariate if birds are sampled during the non-breeding season. Moreover, given that sex also interacts with breeding stage, condition indices should not be collected during the breeding season without detailed knowledge of the reproductive state of the individuals, a suggestion also made by others (Saino *et al.*, 2001).

Scaled mass was the only condition index to vary with age in crimson finches (Table 5). Lack of pervasive effects of age on condition indices is consistent with some studies (Sheridan *et al.*, 2004), but inconsistent with others, which found that age correlates with total plasma protein (Dyer *et al.*, 2010), PCV (through an interaction with sex), haemoglobin and H/L ratios (Norte *et al.*, 2009b). Ultimately, age effects are best tested longitudinally, which we were unable to do because we sampled few individuals across years and within the same breeding stage.

Two condition indices varied significantly between years in crimson finches, including haemoglobin and muscle scores; H/L ratio and fat scores also varied by year, but these trends were not significant after Bonferroni correction. Others have also found that these and additional condition indices vary by year or season, suggesting an environmental effect on condition (Norte *et al.*, 2009b; Vinkler *et al.*, 2010). However, the annual variation we found was not consistent between condition indices. For example, muscle was lowest during the 2009–10 breeding season, but this was an average year for haemoglobin. Haemoglobin, muscle scores, H/L ratio, and fat scores reflect different aspects of the physiological state of the individual, which may explain the inconsistent trends between these condition indices across years.

### Use of condition indices in conservation

We suspect that the year effects we observed are likely to have resulted from differences in environmental conditions between years, for example variation in food supply mediated by weather. It is this type of relationship between individual condition and environmental conditions that is useful in conservation. If the year effect indeed reflects the environment, then the condition indices that show these year effects, i.e. haemoglobin, pectoral muscle scores, and to a lesser extent, H/L ratio and fat scores, may be the most appropriate ones for conservation applications. We documented considerable variability in condition indices attributable to sex, breeding stage, and the interaction between sex and breeding stage, and to a lesser extent age and time of day. This variation must be accounted for, either statistically or via sampling design, in order to interpret condition indices as indicators of environmental conditions. For example, in interpreting differences between two habitats using haemoglobin as an index, one would need to control for the confounding effects of breeding stage and a sex by stage interaction. In such cases, one should not view an absolute value of a given index as a measure of the physiological condition of an individual, but rather the condition of an individual is relative to conspecifics of the same sex, breeding stage, and possibly age. This has previously been suggested for at least some condition indices (e.g. PCV, Fair *et al.*, 2007).

If we assume for the moment that the year effects in our analyses do represent responses to annual variation in environmental conditions, then the two condition indices that exhibited no confounding effects such as sex and stage but did exhibit a year effect, i.e. pectoral muscle score and to a lesser extent H/L ratio, represent better choices for conservation applications. Those condition indices that exhibit several confounding effects but no year effect are the poorest choices, because the link between the environment and the condition index is not as direct and is confounded by other variables. However, our assumption about the source of year effects could be incorrect. Although others have demonstrated that condition indices do vary with habitat or environmental quality (Marra and Holberton, 1998; Owen *et al.*, 2005; Johnson, 2007; Homyack, 2010), we cannot exclude the possibility that some or all of the year effects we observed in our study were a function of something other than environmental variation, such as a cohort effect. Understanding the mechanism of how pectoral muscle scores and H/L ratio are affected by environmental conditions, and what the consequences of that are for individuals and populations, are topics for further research.

Underlying the use of condition indices in conservation is the often implicit assumption that condition and fitness are positively correlated; that is, even if a condition index exhibits a relationship to habitat or environmental conditions, if this relationship has no consequences for survival or reproduction then it does not inform conservation. Indeed, some condition indices have been found to correlate with primary components of fitness, such as reproductive success (mean corpuscular volume, Bearhop *et al.*, 1999; total plasma protein, Gregg *et al.*, 2006) and survival (haemoglobin, Ben-David *et al.*, 2002; and H/L ratio, globulin concentration, and albumin to globulin ratio, Kilgas *et al.*, 2006b). Of the four condition indices that we suggest may be useful in conservation, both haemoglobin and H/L ratio have been found to predict survival (Ben-David *et al.*, 2002; Kilgas *et al.*, 2006b), indicating that they may be particularly useful indices, and we are not aware of any studies that tested whether muscle or fat score relate to fitness. However, the hypothesis that condition indices are meaningful indicators of fitness has been questioned (Dawson and Bortolotti, 1997; Lailvaux and Kasumovic, 2011) and needs to be validated (Stevenson and Woods, 2006; Wikelski and Cooke, 2006; Johnson, 2007; Johnstone *et al.*, 2012b) if condition indices are to be useful tools for conservation.

## References

[1] Ardia DR (2005) Individual quality mediates trade-offs between reproductive effort and immune function in tree swallows. J Anim Ecol 74: 517–524.

[2] Averbeck C (1992) Haematology and blood chemistry of healthy and clinically abnormal great black-backed gulls (*Larus marinus*) and herring gulls (*Larus argentatus*). Avian Pathol 21: 215–223. 18670934 10.1080/03079459208418837

[3] Bearhop S Griffiths R Orr K Furness RW (1999) Mean corpuscular volume (MCV) as a measure of condition in birds. Ecol Lett 2: 352–356.

[4] Ben-David M Blundell GM Blake JE (2002) Post-release survival of river otters: effects of exposure to crude oil and captivity. J Wild Manage 66: 1208–1223.

[5] Birchard GF (1997) Optimal hematocrit: theory, regulation and implications. Am Zool 37: 65–72.

[6] Brown ME (1996) Assessing body condition in birds. In VJ Nolan, ED Ketterson, eds, Current Ornithology, Vol 13. Plenum Press, New York, pp 67–135.

[7] Campbell TW Ellis CK (2007) Avian and Exotic Animal Hematology and Cytology. Blackwell Publishing, Ames.

[8] Carpenter FL (1975) Bird hematocrits: effects of high altitude and strength of flight. *Comp Biochem Physiol A* 50A: 415–417. 10.1016/0300-9629(75)90035-3 234354

[9] Cooke SJ Sack L Franklin CE Farrell AP Beardall J Wikelski M Chown SL (2013) What is conservation physiology? Perspectives on an increasingly integrated and essential science. Conserv Physiol 1: 1–23. 10.1093/conphys/cot001 PMC4732437 27293585

[10] Davis AK Maney DL Maerz JC (2008) The use of leukocyte profiles to measure stress in vertebrates: a review for ecologists. Funct Ecol 22: 760–772.

[11] Dawson RD Bortolotti GR (1997) Variation in hematocrit and total plasma proteins of nestling American Kestrels (*Falco sparverius*) in the wild. Comp Biochem Physiol A 117: 383–390.

[12] de Lope F Møller AP de la Cruz C (1998) Parasitism, immune response and reproductive success in the house martin *Delichon urbica*. Oecologia 114: 188–193. 10.1007/s004420050435 28307931

[13] Dorricott KE Garnett ST (2007) National recovery plan for the white-bellied subspecies of the crimson finch *Neochmia phaeton evangelinae* and the Northern subspecies of the star finch *Neochmia ruficauda clarescens*. Report to the Austrlaian Government Department of the Environment and Water Resources, Canberra. Queensland Parks and Wildlife Service, Brisbane.

[14] Dyer KJ Perryman BL Holcombe DW (2009) Fitness and nutrtional assessment of greater sage grouse (Centrocercus urophasianus) using hematologic and serum chemistry parameters through a cycle of seasonal habitats in northern Nevada. J Zoo Wildl Med 40: 18–28. 19368237 10.1638/2007-0073.1

[15] Dyer KJ Perryman BL Holcombe DW (2010) Site and age class variation of hematologic parameters for female Greater Sage Grouse of northern Nevada. J Wildl Dis 46: 1–12. 20090013 10.7589/0090-3558-46.1.1

[16] Ellenberg U Setiawan AN Cree A Houston DM Seddon PJ (2007) Elevated hormonal stress response and reduced reproductive output in Yellow-eyed penguins exposed to unregulated tourism. Gen Comp Endocrinol 152: 54–63. 17400221 10.1016/j.ygcen.2007.02.022

[17] Ellis RD McWhorter TJ Maron M (2012) Integrating landscape ecology and conservation physiology. Landsc Ecol 27: 1–12.

[18] Fair J Whitaker S Pearson B (2007) Sources of variation in haematocrit in birds. Ibis 149: 535–552.

[19] Fox J (2010) Polycor: polychoric and polyserial correlations. R package version 0.7-8. http://CRAN.R-project.org/package=polycor.

[20] Franklin DC Whitehead PJ Pardon G Matthews J McMahon P McIntyre D (2005) Geographic patterns and correlates of the decline of granivorous birds in northern Australia. Wildl Res 32: 399–408.

[21] Garnett ST Szabo JK Dutson G (2011) The Action Plan for Australian Birds 2010. CSIRO Publishing, Collingwood.

[22] Garvin JC Dunn PO Whittingham LA Steeber DA Hasselquist D (2007) Do male ornaments signal immunity in the common yellowthroat? Behav Ecol 19: 54–60.

[23] Gavett AP Wakeley JS (1986) Blood constituents and their relation to diet in urban and rural House Sparrows. Condor 88: 279–284.

[24] George JW (2001) The usefulness and limitations of hand-held refractometers in veterinary laboratory medicine: an historical and technical review. Vet Clin Path 30: 201–210. 12024303 10.1111/j.1939-165x.2001.tb00432.x

[25] Greenman CG Martin LB II Hau M (2005) Reproductive state but not testosterone reduces immune function in male house sparrows (*Passer domesticus*). Physiol Biochem Zool 78: 60–68. 15702464 10.1086/425194

[26] Gregg MA Dunbar MR Crawford JA Pope MD (2006) Total plasma protein and renesting by Greater Sage-Grouse. J Wildl Manage 70: 472–478.

[27] Harrison GJ Harrison LR (1986) Clinical Avian Medicine and Surgery, Including Aviculture. Saunders, Philadelphia.

[28] Hatch MI Smith RJ Owen JC (2010) Arrival timing and hematological parameters in Gray Catbirds (*Dumetella carolinensis*). J Ornithol 151: 545–552.

[29] Henry M Cosson J-F Pons J-M (2007) Abundance may be a misleading indicator of fragmentation-sensitivity: the case of fig-eating bats. Biol Conserv 139: 462–467.

[30] Higgins PJ Peter JM Cowling SJ (2006) Handbook of Australian, New Zealand & Antarctic Birds: Boatbill to Starlings Part B. Oxford University Press, South Melbourne.

[31] Homyack JA (2010) Evaluating habitat quality of vertebrates using conservation physiology tools. Wildl Res 37: 332–342.

[32] Hõrak P Jenni-Eiermann S Ots I Tegelmann L (1998a) Health and reproduction: the sex-specific clinical profile of great tits (*Parus major*) in relation to breeding. Can J Zool 76: 2235–2244.

[33] Hõrak P Ots I Murumagi A (1998b) Haematological health state indices of reproducing Great Tits: a response to brood size manipulation. Funct Ecol 12: 750–756.

[34] Janin A Léna J-P Joly P (2011) Beyond occurrence: body condition and stress hormone as integrative indicators of habitat availability and fragmentation in the common toad. Biol Conserv 144: 1008–1016.

[35] Johnson MD (2007) Measuring habitat quality: a review. Condor 109: 489–504.

[36] Johnstone CP Lill A Reina RD (2012a) Does habitat fragmentation cause stress in the agile antechinus? A haematological approach. J Comp Physiol B 182: 139–155. 21710385 10.1007/s00360-011-0598-7

[37] Johnstone CP Reina RD Lill A (2012b) Interpreting indices of physiological stress in free-living vertebrates. J Comp Physiol B 182: 861–879. 22415475 10.1007/s00360-012-0656-9

[38] Kasprzak M Hetmański T Kulczykowska E (2006) Changes in hematological parameters in free-living pigeons (*Columba livia f. urbana*) during the breeding cycle. J Ornithol 147: 599–604.

[39] Kilgas P Mänd R Mägi M Tilgar V (2006a) Hematological parameters in brood-rearing great tits in relation to habitat, multiple breeding and sex. Comp Biochem Physiol A 144: 224–231. 10.1016/j.cbpa.2006.02.038 16616538

[40] Kilgas P Tilgar V Mand R (2006b) Hematological health state indices predict local survival in a small passerine bird, the Great Tit (*Parus major*). Physiol Biochem Zool 79: 565–572. 16691522 10.1086/502817

[41] Kitaysky AS Wingfield JC Piatt JF (1999) Dynamics of food availability, body condition, and physiological stress response in breeding Black-legged Kittiwakes. Funct Ecol 13: 577–584.

[42] Lailvaux SP Kasumovic MM (2011) Defining individual quality over lifetimes and selective contexts. Proc R Soc Lond B Biol Sci 278: 321–328. 10.1098/rspb.2010.1591 PMC3013420 20861050

[43] McNamara JM Houston AI (1990) The value of fat reserves and the tradeoff between starvation and predation. Acta Biotheor 38: 37–61. 2109917 10.1007/BF00047272

[44] Maron M Goulding W Ellis RD Mohd-Taib F-S (2012) Distribution and individual condition reveal a hierarchy of habitat suitability for an area-sensitive passerine. Biodivers Conserv 21: 2509–2523.

[45] Marra PP Holberton RL (1998) Corticosterone levels as indicators of habitat quality: effects of habitat segregation in a migratory bird during the non-breeding season. Oecologia 116: 284–292. 10.1007/s004420050590 28308538

[46] Maute KL (2011) Variation in the health of tropical finches in relation to conservation status, season and land tenure. Doctor of Philosophy Dissertation, University of Wollongong, Wollongong.

[47] Milenkaya O Legge S Walters JR (2011) Breeding biology and life-history traits of an Australasian tropical granivore, the Crimson Finch (*Neochmia phaeton*). Emu 111: 312–320.

[48] Morton ML (1994) Hematocrits in montane sparrows in relation to reproductive schedule. Condor 96: 119–126.

[49] Neto JM Gosler AG (2009) Variation in body condition of breeding Savi's Warblers *Locustella luscinioides*: the reproductive stress and flight adaptation hypothesis revisited. J Ornithol 151: 201–210.

[50] Norte AC Araújo PM Sampaio HL Sousa JP Ramos JA (2009a) Haematozoa infections in a Great Tit *Parus major* population in Central Portugal: relationships with breeding effort and health. Ibis 151: 677–688.

[51] Norte AC Ramos JA Sousa JP Sheldon BC (2009b) Variation of adult Great Tit *Parus major* body condition and blood parameters in relation to sex, age, year and season. J Ornithol 150: 651–660.

[52] Ots I Hõrak P (1998) Health impact of blood parasites in breeding great tits. Oecologia 116: 441–448. 10.1007/s004420050608 28307512

[53] Owen JC Sogge MK Kern MD (2005) Habitat and sex differences in physiological condition of breeding Southwestern Willow Flycatchers (*Empidonax trailliextimus*). Auk 122: 1261–1270.

[54] Peig J Green AJ (2009) New perspectives for estimating body condition from mass/length data: the scaled mass index as an alternative method. Oikos 118: 1883–1891.

[55] Pravosudov VV Grubb TC (1997) Management of fat reserves and food caches in tufted titmice (*Parus bicolor*) in relation to unpredictable food supply. Behav Ecol 8: 332–339.

[56] Pyle P (1997) Identification Guide to North American Birds Part I: Columbidae to Ploceidae. Slate Creek Press, Bolinas, CA.

[57] R Development Core Team (2012) R: A language and environment for statistical computing. R Foundation for Statistical Computing, Vienna, Austria.

[58] Rogers C (2005) Food limitation among wintering birds. In R Greenberg, PP Marra, eds, Birds of Two Worlds: The Ecology and Evolution of Migration. The Johns Hopkins University Press, Baltimore, pp 106–113.

[59] Rosenthal KL (2000) Avian protein disorders. In AM Fudge, ed, Laboratory Medecine: Avian and Exotic Pets. W.B. Saunders Company, Philadelphia, pp 171–173.

[60] Saino N Cuervo JJ Ninni P deLope F Møller AP (1997) Haematocrit correlates with tail ornament size in three populations of the Barn Swallow (*Hirundo rustica*). Funct Ecol 11: 604–610.

[61] Saino N Martinelli R Møller AP (2001) Immunoglobulin plasma concentration in relation to egg laying and mate ornamentation of female barn swallows (*Hirundo rustica*). J Evol Biol 14: 95–109. 10.1046/j.1420-9101.2001.00252.x 29280578

[62] Schoech SJ Bowman R (2003) Does differential access to protein influence differences in timing of breeding of Florida scrub-jays (*Aphelocoma coerulescens*) in suburban and wildland habitats? Auk 120: 1114–1127.

[63] Semeniuk CAD Bourgeon S Smith SL Rothley KD (2009) Hematological differences between stingrays at tourist and non-visited sites suggest physiological costs of wildlife tourism. Biol Conserv 142: 1818–1829.

[64] Sepp T Sild E Hõrak P (2010) Hematological condition indexes in greenfinches: effects of captivity and diurnal variation. Physiol Biochem Zool 83: 276–282. 20078208 10.1086/648580

[65] Shallin Busch D Robinson WD Robinson TR Wingfield JC (2011) Influence of proximity to a geographical range limit on the physiology of a tropical bird. J Anim Ecol 80: 640–649. 21219328 10.1111/j.1365-2656.2010.01791.x

[66] Sheridan JA Beissinger SR Hughes CR (2004) Weak association between measures of health and reproductive success in green-rumped parrotlets (*Forpus passerinus*) in Venezuela. Auk 121: 717–725.

[67] Stevenson RD Woods WA Jr (2006) Condition indices for conservation: new uses for evolving tools. Integr Comp Biol 46: 1169–1190. 21672816 10.1093/icb/icl052

[68] Suorsa P Huhta E Nikula A Nikinmaa M Jäntti A Helle H Hakkarainen H (2003) Forest management is associated with physiological stress in an old-growth forest passerine. Proc R Soc Lond B Biol Sci 270: 963–969. 10.1098/rspb.2002.2326 PMC1691328 12803912

[69] Szabó K Szalmás A Liker A Barta Z (2002) Effects of haematophagous mites on nestling house sparrows (*Passer domesticus*). Acta Parasitol 47: 318–322.

[70] Tomkins JL Radwan J Kotiaho JS Tregenza T (2004) Genic capture and resolving the lek paradox. Trends Ecol Evol 19: 323–328. 16701278 10.1016/j.tree.2004.03.029

[71] Velguth KE Payton ME Hoover JP (2010) Relationship of hemoglobin concentration to packed cell volume in avian blood samples. J Avian Med Surg 24: 115–121. 20806656 10.1647/2008-042.1

[72] Vinkler M Schnitzer J Munclinger P Votýpka J Albrecht T (2010) Haematological health assessment in a passerine with extremely high proportion of basophils in peripheral blood. J Ornithol 151: 841–849.

[73] Wagner EC Prevolsek JS Wynne-Edwards KE Williams TD (2008) Hematological changes associated with egg production: estrogen dependence and repeatability. J Exp Biol 211: 400–408. 18203996 10.1242/jeb.011205

[74] Walker BG Boersma PD Wingfield JC (2005) Field endocrinology and conservation biology. Integr Comp Biol 45: 12–18. 21676739 10.1093/icb/45.1.12

[75] Wasser SK Bevis K King G Hanson E (1997) Noninvasive physiological measures of distubance in the northern spotted owl. Conserv Biol 11: 1019–1022.

[76] Wikelski M Cooke SJ (2006) Conservation physiology. Trends Ecol Evol 21: 38–46. 16701468 10.1016/j.tree.2005.10.018

[77] Williams TD Challenger WO Christians JK Evanson M Love OP Vezina F (2004) What causes the decrease in haematocrit during egg production? Funct Ecol 18: 330–336.

[78] Woinarski JCZ Legge S Fitzsimons JA Traill BJ Burbidge AA Fisher A Firth RSC Gordon IJ Griffiths AD Johnson CN (2011) The disappearing mammal fauna of northern Australia: context, cause, and response. Conserv Lett 4: 192–201.

